# Relevance of plasma lenvatinib concentrations and endogenous urinary cytochrome P450 3A activity biomarkers in clinical practice

**DOI:** 10.1002/prp2.1241

**Published:** 2024-07-11

**Authors:** Masaki Kumondai, Reika Ogawa, Nagomi Hayashi, Yurika Ishida, Hanae Oshikiri, Yuji Sato, Masafumi Kikuchi, Yu Sato, Toshihiro Sato, Masamitsu Maekawa, Nariyasu Mano

**Affiliations:** ^1^ Department of Pharmaceutical Sciences Tohoku University Hospital Sendai Japan; ^2^ Faculty of Pharmaceutical Sciences Tohoku University Sendai Japan

**Keywords:** biomarkers, cytochrome P450 3A, lenvatinib, therapeutic drug monitoring

## Abstract

Lenvatinib (LEN), a multitarget tyrosine kinase inhibitor used in various cancer treatments, is mainly metabolized by cytochrome P450 3A (CYP3A) enzymes. The importance of therapeutic drug monitoring (TDM) in patients administered LEN has been proposed. Although some biomarkers of endogenous CYP3A activity have been reported, their utility in dosage adjustments has not been well evaluated. This study investigated the correlation between plasma LEN concentrations and endogenous urinary CYP3A biomarkers in clinical practice. Concentrations of plasma LEN (*N* = 225) and CYP3A biomarkers (cortisol, 6β‐hydroxycortisol, deoxycholic acid, and 1β‐hydroxydeoxycholic acid) in urine (*N* = 214) from 20 patients (hepatocellular carcinoma, *N* = 6; thyroid cancer, *N* = 3; endometrial cancer, *N* = 8; and renal cell carcinoma, *N* = 3) collected for consultation for up to 1 year were evaluated using liquid chromatography–tandem mass spectrometry. Moreover, plasma trough LEN concentrations were predicted using a three‐compartment model with linear elimination for outpatients administered LEN before sample collection. Moderate correlations were observed between the quantified actual concentrations and the predicted trough concentrations of LEN, whereas there was no correlation with endogenous urinary CYP3A biomarkers. The utility of endogenous urinary CYP3A biomarkers could not be determined. However, TDM for outpatients administered orally available medicines may be predicted using a nonlinear mixed effect model (NONMEM). This study investigated the utility of endogenous urinary CYP3A biomarkers for personalized medicine and NONMEM for predicting plasma trough drug concentrations. These findings will provide important information for further clinical investigation and detailed TDM.

## Graphical abstract

Colleration between measured and predicted plasma lenvatinib concentrations.

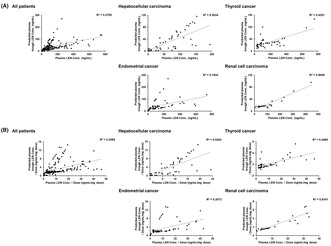

**Abbreviations**
1β‐OHDCA: 1β‐hydroxydeoxycholic acid
6β‐OHC: 6β‐hydroxycortisol
C: cortisol
CYP3A: cytochrome P450 3A
DCA: deoxycholic acid
LC–MS/MS: liquid chromatography–tandem mass spectrometry
LEN: lenvatinib
NONMEM: nonlinear mixed effect model
TDM: therapeutic drug monitoring

## 1 INTRODUCTION

Lenvatinib (LEN), an orally available multitargeting tyrosine kinase inhibitor, is used as an anticancer drug in patients with various cancers, including hepatocellular carcinoma,^1^, ^2^ thyroid cancer,^3^ thymic carcinoma,^4^ endometrial cancer,^5^ and renal cell carcinoma.^5^ The approved initial dose varies for cancer types. Namely, LEN dosages of 8 or 12 mg/day for hepatocellular carcinoma, 20 mg/day for thyroid and endometrial cancer, and 24 mg/day for thymic and renal cell carcinoma are required for cancer therapy. Therapeutic drug monitoring (TDM), whose utility is routinely investigated for various drugs including anticancer drugs in clinical practice, is clinically important as relatively strong correlations between plasma multikinase inhibitor concentrations and drug efficacy or toxicity in patients have been reported.^6^, ^7^, ^8^ For instance, the optimal plasma trough LEN concentration in patients with thyroid cancer is expected to be from 42 to 88 ng/mL for optimal response, whereas drug toxicity may occur at more than 88 ng/mL.^7^ In contrast, the expected target trough concentration of LEN in patients with hepatocellular carcinoma was reported to be between 36.8 and 71.4 ng/mL for maintaining disease control status with reduced toxicity.^8^ Various adverse effects caused by LEN, including hypertension, diarrhea, proteinuria, and fatigue, are known to occur frequently, resulting in dosage reduction or drug suspension.^9^, ^10^ However, determining the optimal LEN dose prior to drug treatment is difficult.

The human cytochrome P450 3A (CYP3A) family, which includes CYP3A4 and CYP3A5, contains the primary drug metabolism enzymes.^11^, ^12^ Importantly, both CYP3A4 and CYP3A5 contribute to the metabolism of 30% of medications, including various kinase inhibitors.^13^, ^14^ For the improvement of personalized medicine, the prediction of CYP3A4 and CYP3A5 activities would play an important role; however, this remains a challenge as various genetic and environmental factors, including CYP3A inducers and inhibitors, drug–drug interactions, and genetic polymorphisms, affect the interindividual differences in CYP3A activity.^15^, ^16^, ^17^, ^18^, ^19^, ^20^ Interindividual CYP3A activity may be used to implement personalized medicine and individualized dosing regimens.

LEN is mainly metabolized to *N*‐oxide and *O*‐desmethyl LEN by CYP3A4.^21^ Interestingly, although the metabolism of numerous drugs overlaps with that of CYP3A4 and CYP3A5, LEN metabolism via CYP3A5 does not occur in vitro.^22^ The effect of CYP3A4 activity on plasma LEN concentration has been reported.^23^, ^24^ The dose‐adjusted plasma LEN concentrations in patients carrying the CYP3A4*1G allele were significantly lower than those in patients carrying wild‐type *CYP3A4*.^24^ Thus, LEN dosage adjustment, considering CYP3A4 enzymatic activity, may provide a more precise anticancer therapy.

**FIGURE 1:**
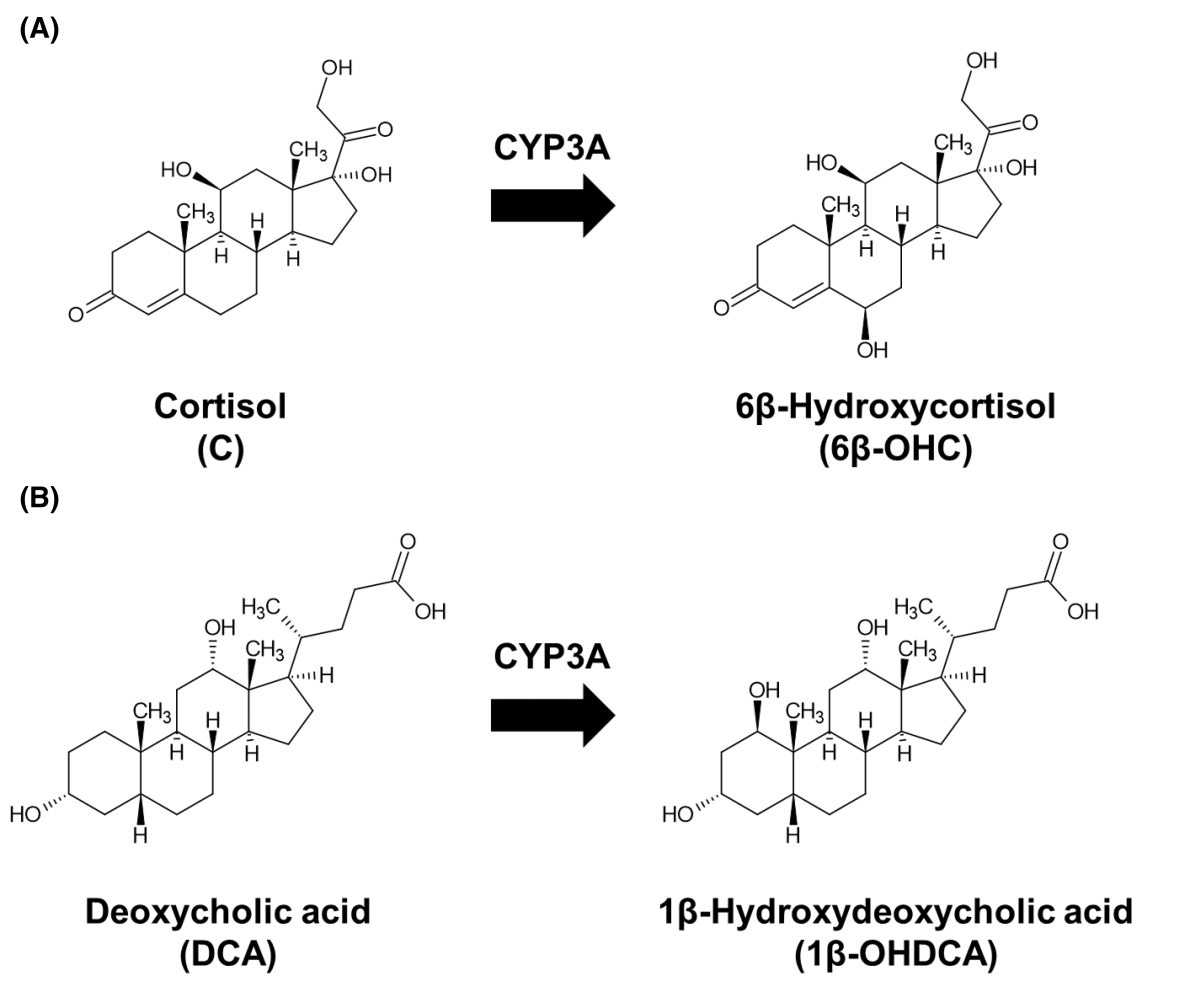
Metabolic pathways from cortisol (C) to 6β‐hydroxycortisol (A) and deoxycholic acid to 1β‐hydroxydeoxycholic acid (B) catalyzed by cytochrome P450 3A.

As for CYP3A activity prediction, endogenous biomarkers such as 1β‐hydroxydeoxycholic acid (1β‐OHDCA),^25^, ^26^, ^27^ 6β‐hydroxycortisol (6β‐OHC),^28^, ^29^, ^30^ and 4β‐hydroxycholesterol,^31^, ^32^, ^33^ and these substrates in plasma or urine are reported as CYP3A activity biomarkers (Figure 1). Both 1β‐OHDCA and 6β‐OHC may represent better indicators of actual CYP3A activity during sample collection due to having a shorter half‐life than 4β‐hydroxycholesterol.^31^ Moreover, 1β‐OHDCA and 6β‐OHC, as well as these substrates, can be noninvasively quantified because both exist in urine. We recently developed an analytical method based on liquid chromatography–tandem mass spectrometry (LC–MS/MS) that can simultaneously quantify urinary endogenous CYP3A biomarkers: 1β‐OHDCA, deoxycholic acid (DCA), 6β‐OHC, and cortisol (C).^34^ However, there are currently no defined biomarkers reflecting CYP3A activity.

This study aimed to investigate the clinical usefulness of urinary endogenous CYP3A biomarkers by evaluating the correlation between plasma LEN concentrations and urinary biomarker concentrations. Moreover, a nonlinear mixed effect model (NONMEM) was applied to trough LEN concentrations in the plasma without any additional burden on the patients.

## 2 MATERIALS AND METHODS

### 2.1 Chemical and reagents

Reagents were purchased from the following sources: 6β‐OHC, 6β‐hydroxycortisol‐^2^H_4_, and 1β‐OHDCA (Santa‐Cruz Biotechnology, Dallas, TX, USA); C, DCA, deoxycholic acid‐^2^H_4_, cholic acid‐^2^H_4_, 25% ammonia solution, sulfatase from *Clostridium perfringens*, and choloylglycine hydrolase from *Clostridium perfringens* (Sigma‐Aldrich, St. Louis, MO, USA); cortisol‐^13^C_3_ (Cambridge Isotope Laboratories, Tewksbury, MA, USA); LEN (Cayman Chemical Company, Ann Arbor, MI, USA); lenvatinib‐^2^H_5_ (Alsachim, Illkirch‐Graffenstaden, France); and β‐glucuronidase/arylsulfatase (F. Hoffmann‐La Roche, Ltd., Basel, Switzerland). All other chemicals and reagents were of the highest commercially available quality.

### 2.2 Subjects

Inpatients and outpatients who started administration of LEN for treating hepatocellular carcinoma, thyroid cancer, endometrial cancer, or renal cell carcinoma between April 2022 and September 2023 were recruited from Tohoku University Hospital (Sendai, Japan). The characteristics of the participants are summarized in Table 1. Plasma samples were collected after taking LEN but not trough point and immediately stored at −80°C until use. Urine samples were collected on the same day as blood samples, within 60 min before or after blood sampling. Plasma and urine samples from the same patients were collected continuously for up to 1 year.

**TABLE 1:** Background characteristics and therapy schedules for each participant.

Number	Sex	Age	Cancer type	Dose amount (mg/day)	Administration	Event
1	Male	84	Hepatocellular carcinoma	8	Everyday	
					5‐day on/2‐day off (day 56–)	Fatigue
2	Female	62	Thyroid cancer	24	Everyday	
					5‐day on/2‐day off (day 16–)	Hypertension
				20 (day 25–217)		Fatigue
				14 (day 218–)		
3	Male	70	Hepatocellular carcinoma	12	5‐day on/2‐day off	
4	Female	71	Endometrial cancer	20	Everyday	
					Suspension (day 13–23, 49–67, 87–99, 149–178)	Hypertension
				14 (day 24–48, 68–86)		Diarrhea
				10 (day 100–148, 179–254)		Hypothyroidism
					Suspension (day 255–)	Nausea
						Fatigue
5	Female	60	Endometrial cancer	20	Everyday	
					Suspension (day 100–112, 205–226, 248–257)	Hypertension
				14 (day 113–204)		
				10 (day 227–247, 258–)		
6	Female	46	Renal cell carcinoma	20	Everyday	
				10 (day 16–)		Hypertension
7	Female	69	Endometrial cancer	20 (day 1–13, 23–44, 109–)	Everyday	
					Suspension (day 14–22)	Bullous Pemphigoid
						by pembrolizumab
					Suspension (day 45–108)	Hypertension
						Hypothyroidism
8	Female	78	Endometrial cancer	14	Everyday	
					Suspension (day 24–44, 160–)	Fatigue
						Hypothyroidism
						Urine protein
				10 (day 45–159)		
9	Female	74	Thyroid cancer	24	Everyday	
					Suspension (day 16–24, 30–45, 51–61)	Hand foot syndrome
				20 (day 25–29)		
				14 (day 46–50)		
				10 (day 62–112)		
					5‐day on/2‐day off (day 86–134)	
				8 (day 113–)		
					4‐day on/3‐day off (day 141–)	
10	Male	77	Hepatocellular carcinoma	8	Everyday	
					Suspension (day 50–57)	Ophthalmic surgery
11	Female	73	Endometrial cancer	20	Everyday	
					Suspension (day 16–22, 38–43, 120–)	Pancytopenia
				14 (day 23–37)		
				10 (day 44–119)		
12	Female	66	Endometrial cancer	20	Everyday	
					Suspension (day 19–22)	Hypothyroidism
				14 (day 23–144)		
					Suspension (day 145–163)	Diarrhea
				10 (day 164–208)		
					Suspension (day 209–230)	Hand foot syndrome
				8 (day 231–304)		
					Suspension (day 305–307)	Fatigue
				4 (day 308–)		
13	Female	64	Thyroid cancer	24	Everyday	
					5‐day on/2‐day off (day 15–)	Hypertension
						Fatigue
				20 (day 24–231)		
				14 (day 232–)		Hand foot syndrome
14	Female	78	Endometrial cancer	20	Everyday	
				14 (day 8–15)		Hypertension
					Suspension (day 16–21)	Fatigue
						Hypothyroidism
				10 (day 22–35)		
					Suspension (day 36–)	Skin rash
15	Female	64	Endometrial cancer	20	Everyday	
					Suspension (day 32–63)	Hand foot syndrome
				14 (day 64–)		
16	Male	80	Hepatocellular carcinoma	8	Everyday	
					Suspension (day 21–)	Transcatheter arterial chemo‐embolization (day 23)
17	Female	65	Renal cell carcinoma	20	Everyday	
				14 (day 8–21)		Hypertension
				10 (day 22–35)		Fatigue
				4 (day 36–116)		Urine protein
					Suspension (day 117–)	Urine protein
18	Male	76	Hepatocellular carcinoma	8	Everyday	
					Suspension (day 48–70, 78–97)	Fatigue
					5‐day on/2‐day off (day 98–)	
19	Male	53	Hepatocellular carcinoma	8	Everyday	
					Suspension (day 36–40)	Urine protein
					5‐day on/2‐day off (day 41–49)	
					Suspension (day 50–)	Chemotherapy change
20	Male	73	Renal cell carcinoma	10	Everyday	
					Suspension (day 170–)	Urine protein

### 2.3 Quantification of plasma LEN concentrations

The plasma LEN concentrations were quantified using previously validated, conventional methods.^2^ Briefly, 160 μL of acetonitrile–methanol (9:1, v/v) solution containing 0.8 ng of lenvatinib‐^2^H_5_ was added to 40 μL of the plasma samples. The mixture was vortexed and centrifuged at 14 000 *g* for 10 min at 4°C. After centrifugation, 2 μL of supernatant was injected into the LC–MS/MS for quantification. An LC–MS/MS system was used in positive ion detection mode at the electrospray ionization interface (LCMS8050; Shimadzu, Kyoto, Japan). For separation, the NexeraX2 ultra‐high‐performance liquid chromatography system (Shimadzu) was used with a YMC‐Triart C18 metal‐free column (2.1 mm i.d. × 50 mm, 3 μm; YMC, Kyoto, Japan) maintained at 40°C. Mobile phases were prepared using deionized water containing 20 mM ammonium formate (pH 3.6) as eluent A and methanol containing 20 mM ammonium formate (pH 3.6) as eluent B. The gradient program was as follows: elution was initiated using 5% eluent B for 1 min, followed by a linear gradient of 100% eluent B for 1–3 min. A linear gradient of 100% eluent B was maintained for 1 min and then immediately returned to the initial conditions, which were maintained for 1 min until the end of the run, at a flow rate of 0.45 mL/min. Quantification analyses were performed in the selected reaction–monitoring mode where ions transitioning from the precursor into product ions were monitored: mass‐to‐charge ratio (*m/z*) 427.2 → 370.0 for LEN (Q1 pre‐bias, −14 V; collision energy, −27 V; and Q3 pre‐bias, −28 V) and *m/z* 432.2 → 370.1 for lenvatinib‐^2^H_5_ (Q1 pre‐bias, −25 V; collision energy, −28 V; and Q3 pre‐bias, −26 V). The optimized mass spectrometry settings were as follows: probe voltage, 4000 V; interface temperature, 300°C; desolvation line temperature, 250°C; block heater temperature, 400°C; nebulizing gas flow, 3 L/min; drying gas flow, 10 L/min; and heating gas flow, 10 L/min. Standard curves for LEN were constructed within the range of 1–4000 ng/mL using metabolite standards. The lower limit of quantification was set at 1 ng/mL,^3^ considering previously reported plasma trough LEN concentrations.^7^, ^8^

### 2.4 Plasma LEN concentration prediction

The chronological plasma LEN concentrations were predicted using the NONMEM system (ICON Clinical Research, PA, USA). First, each parameter from a previously published three‐compartment model with linear elimination that utilized population pharmacokinetics data from 15 clinical studies was set in a text file.^35^ Specifically, plasma trough LEN concentrations were predicted by considering the following information: body weight, alkaline phosphatase value, whether any CYP3A4 inhibitors were co‐administered, LEN dosage, LEN‐taking time, and LEN concentrations in plasma. Each dataset was constructed with medication status and blood LEN concentration for 7 days (more than four to five half‐lives) before and after blood collection. The approximate daily dosing times were confirmed, and the relationship with blood sampling time was summarized and used for plasma LEN concentration prediction.

### 2.5 Urinary CYP3A biomarker quantification

The concentrations of urinary biomarkers (1β‐OHDCA, DCA, 6β‐OHC, and C) were quantified as previously validated methods.^34^ Briefly, 100 μL urine was deproteinized by mixing with 900 μL acetonitrile containing internal standards (10 ng deoxycholic acid‐^2^H_4_, 20 ng cholic acid‐^2^H_4_, 2 ng cortisol‐^13^C_3_, and 25 ng 6β‐hydroxycortisol‐^2^H_4_). The supernatant, after centrifugation, was vacuum‐dried. The dried powder was added to hydrolysis enzymes (5 U choloylglycine hydrolase and 10 μL β‐glucuronidase/arylsulfatase) dissolved in 100 μL of 0.1 M Tris–HCl (pH 5.0). The mixture was incubated at 37°C overnight. After adding 300 μL acetonitrile, the centrifugated supernatant was vacuum‐dried at 40°C and dissolved in 50 μL water/methanol/acetonitrile (2:1:1, v/v/v). Samples (5 μL) were analyzed and quantified using a previously validated LC–MS/MS method.^34^

### 2.6 Nomenclature statement

Key protein targets and ligands in this article are hyperlinked to corresponding entries in http://www.guidetopharmacology.org, the common portal for data from the IUPHAR/BPS Guide to PHARMACOLOGY,^36^ and are permanently archived in the Concise Guide to PHARMACOLOGY 2023/24.^37^, ^38^

## 3 RESULTS

**FIGURE 2:**
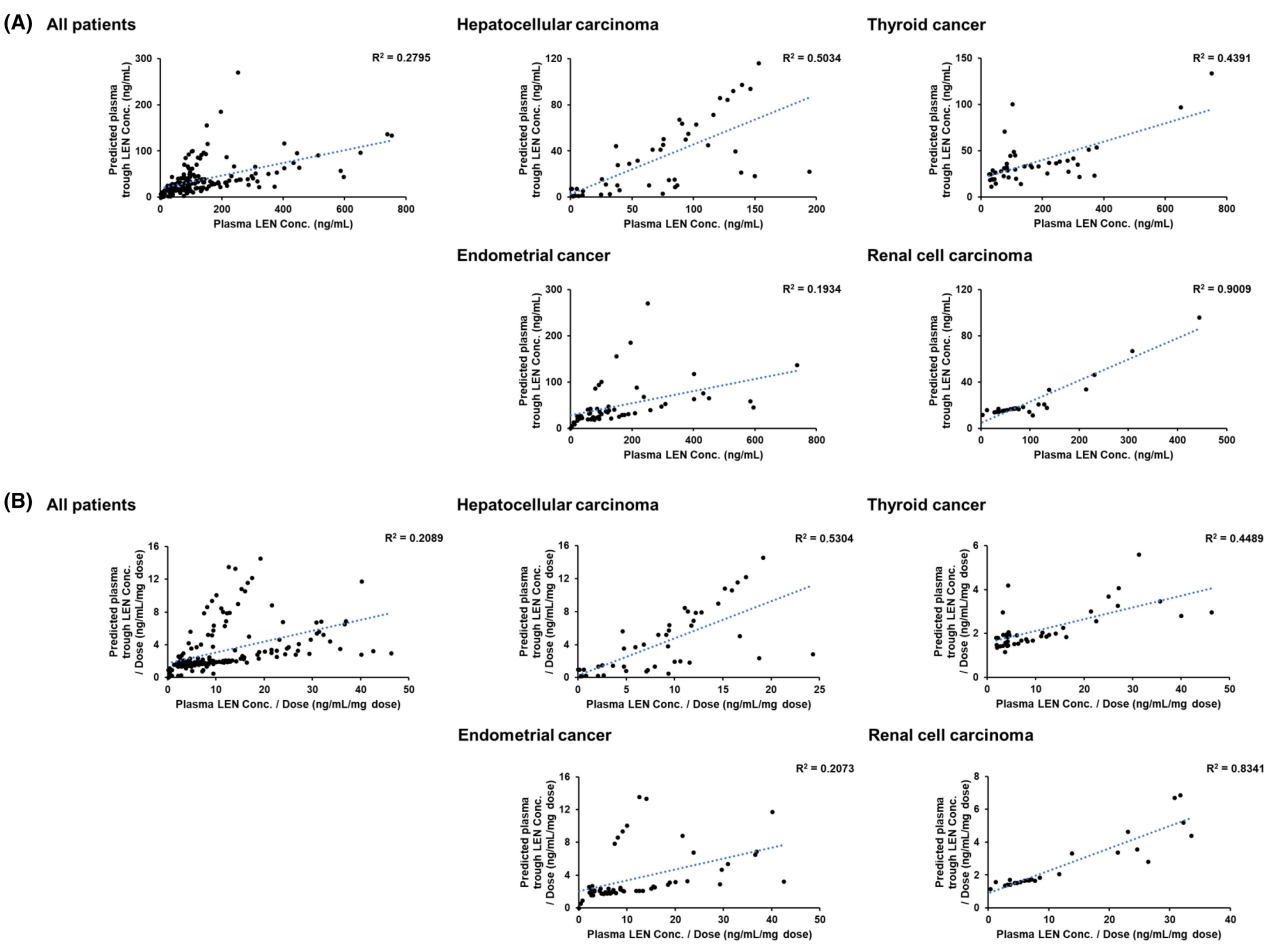
Correlation with plasma lenvatinib (LEN) concentrations for all patients and patients with each cancer type. (A) Predicted plasma trough LEN concentrations and (B) predicted plasma trough LEN concentrations per LEN dose.

Plasma (*N* = 225) and urine samples (*N* = 214) were collected from 20 patients (female/male ratio, 7/13). The therapy schedules for the 20 patients are listed in Table 1. No patients were administrated any CYP3A4 inhibitors. All patients with endometrial or renal cell carcinoma were treated with pembrolizumab. None of the patients with hepatocellular carcinoma were treated with transcatheter arterial chemoembolization during the investigation period, whereas two patients were hepatitis B virus carriers (1 and 19 in Table 1). Plasma samples (*N* = 46) were confirmed to have no remaining LEN because of the LEN suspension period or nonadherence, and these results were excluded from further analysis. Plasma LEN concentrations ranged from <1 to 751.9 ng/mL in samples (*N* = 179) collected while taking LEN administration but not the trough point. Plasma trough LEN concentrations were predicted using the NONMEM program. The approximate LEN administration times and accurate plasma collection times used for the predictions are listed in the Table S1. As shown in Figure 2, moderate correlations (generally defined as .16 ≤ *R* ^2^ < .49) were observed between the predicted plasma trough LEN concentrations and plasma LEN concentrations at the time of blood collection and the concentration per dose ratio when analyzing all patients (*R* ^2^ = .2795 and .2089, respectively). Furthermore, the correlations were moderate or strong (generally defined as .49 ≤ *R* ^2^), except for endometrial cancer, when the results were classified according to cancer type (Figure 2).

**FIGURE 3:**
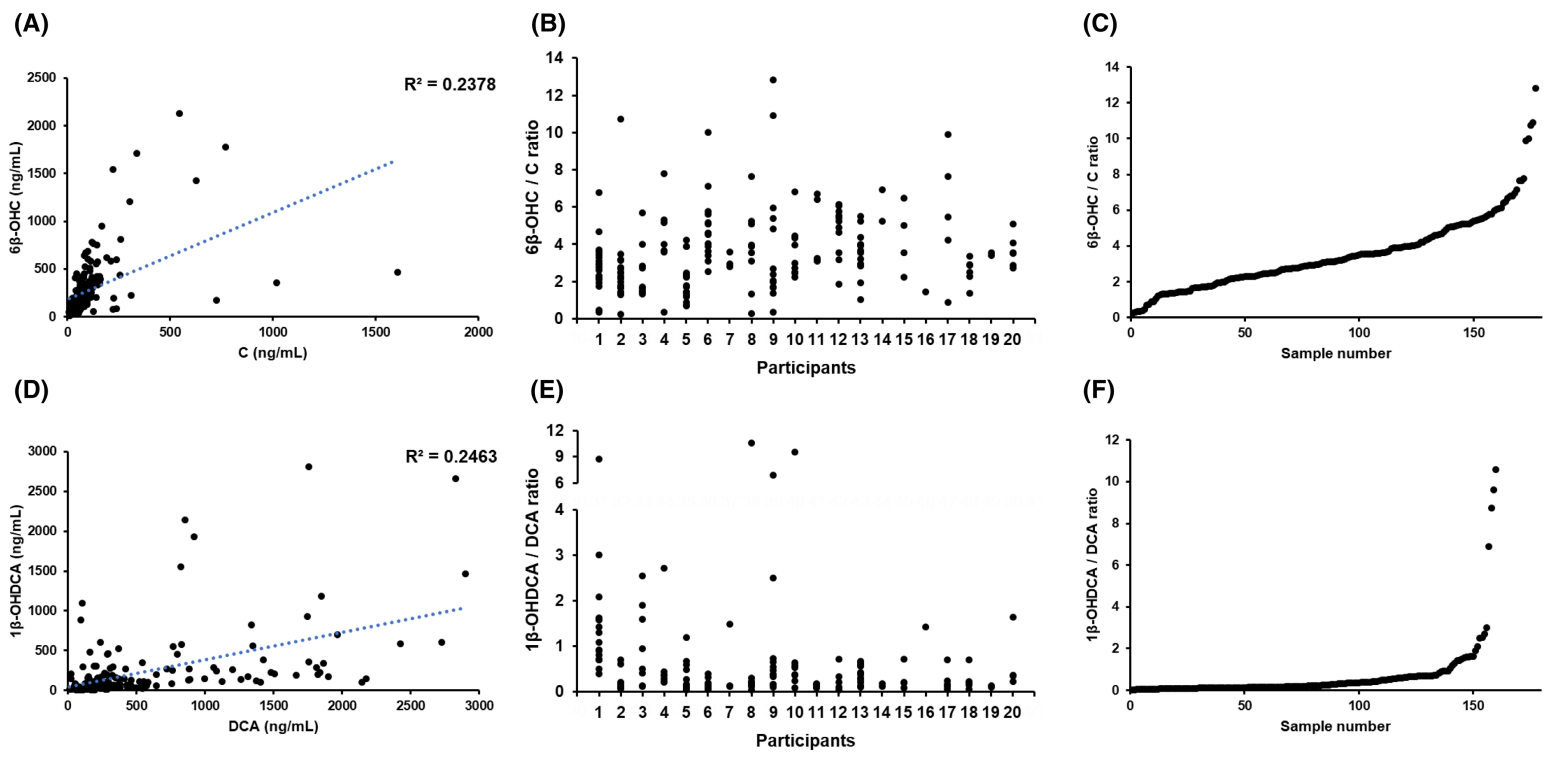
Correlation between urinary concentrations of cortisol (C) and 6β‐hydroxycortisol (6β‐OHC) (A). Intra‐ and interindividual differences in 6β‐OHC/C ratio (B and C, respectively). Correlation between urinary concentrations of deoxycholic acid (DCA) and 1β‐hydroxydeoxycholic acid (1β‐OHDCA) (D). Intra‐ and interindividual differences in 1β‐OHDCA/DCA ratio (E and F, respectively).

**FIGURE 4:**
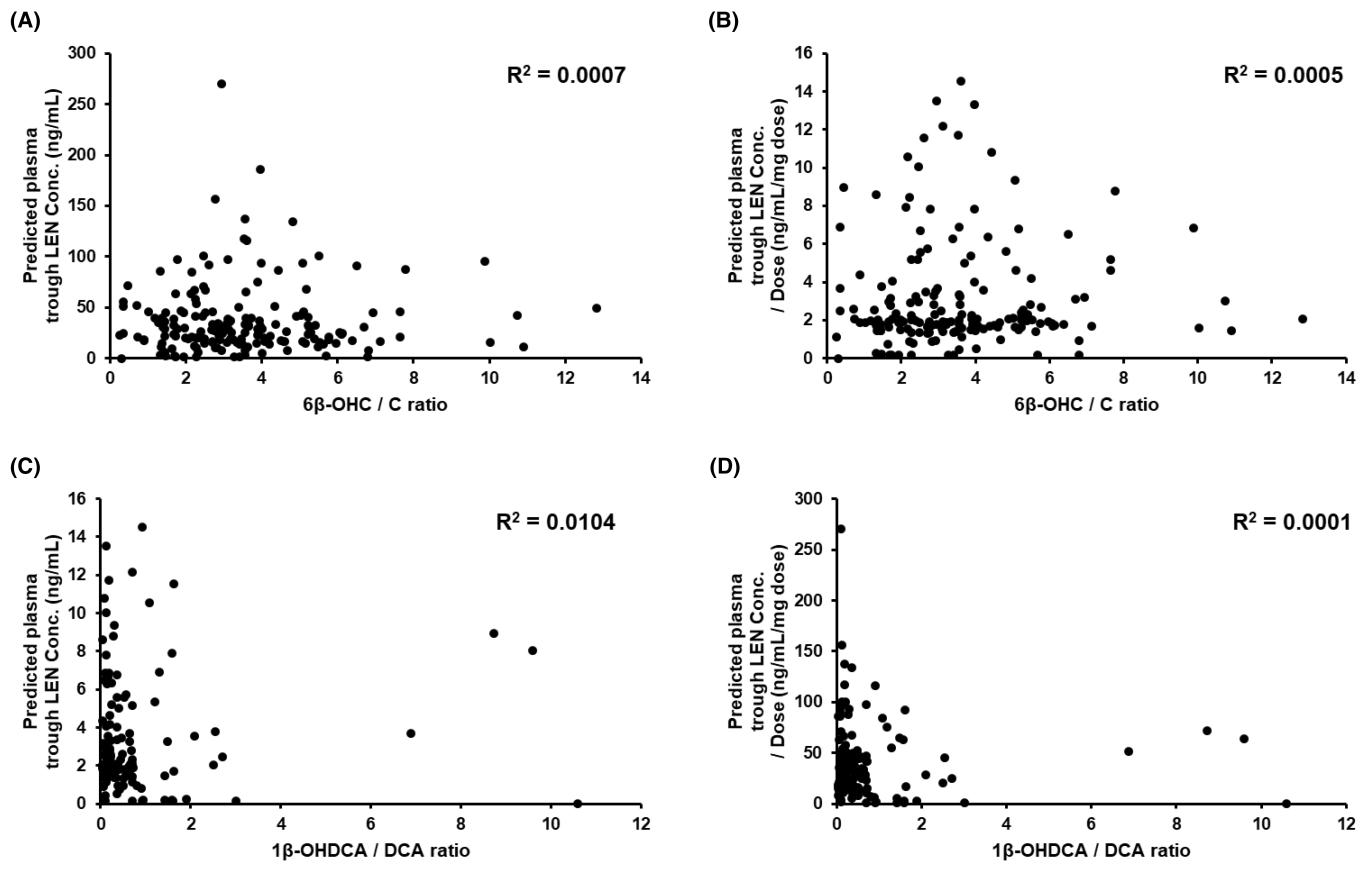
Correlation between 6β‐hydroxycortisol (6β‐OHC)/cortisol (C) ratio and predicted plasma trough lenvatinib (LEN) concentrations (A) and predicted plasma trough LEN concentrations per dose (B). Correlation between 1β‐hydroxydeoxycholic acid (1β‐OHDCA)/deoxycholic acid (DCA) ratio and predicted plasma trough LEN concentrations (C) and predicted plasma trough LEN concentrations per dose (D).

In almost all samples, urinary CYP3A biomarkers were quantified within a calibration standard concentrations range of 3–3000 ng/mL for each analyte (C, *N* = 213; 6β‐OHC, *N* = 213; DCA, *N* = 197; and 1β‐OHDCA, *N* = 210). After excluding the samples outside the calibration standard range, further analysis was performed. As shown in Figure 3A, a moderate correlation between urinary concentrations of C and 6β‐OHC was recognized (*R* ^2^ = .2378). However, the concentration ratios varied intra‐ and interindividually (Figure 3B,C). Similarly, urinary concentrations of DCA and 1β‐OHDCA also observed a moderate correlation, as shown in Figure 3D (*R* ^2^ = .2463). The 1β‐OHDCA/DCA ratios were relatively varied intra‐ and interindividually (Figure 3E,F). Furthermore, the correlations between plasma LEN concentrations and the concentrations of urinary CYP3A biomarkers were analyzed using the values of the samples for which both parameters were available for comparison. There was no correlation between the ratio of CYP3A biomarker concentrations and predicted plasma trough LEN concentrations (Figure 4).

## 4 DISCUSSION

LEN, which is mainly catalyzed by CYP3A, is used to treat various cancers, including hepatocellular carcinoma, thyroid cancer, endometrial cancer, and renal cell carcinoma.^1^, ^2^, ^3^, ^4^, ^5^, ^21^ A therapeutic window for LEN treatment has been suggested for some cancer patients; therefore, chronological monitoring of LEN concentrations in the plasma is important.^7^, ^8^ Endogenous CYP3A biomarkers, which have been used to predict enzymatic CYP3A activity and dosage adjustment, may be applicable for the drug therapy management of LEN by considering its metabolism. This study evaluated the efficacies of measuring endogenous urinary CYP3A biomarkers (C, 6β‐OHC, DCA, and 1β‐OHDCA) compared to plasma LEN concentrations. Moreover, plasma trough LEN concentrations were predicted using NONMEM, the gold standard software for population pharmacokinetics and pharmacokinetic‐pharmacodynamic modeling.^39^

This study protocol was designed to be noninvasive and to reduce the burden on outpatients. We focused on the NONMEM program and endogenous urinary CYP3A biomarkers. NONMEM is widely used for drug development, including in clinical trial simulations.^40^, ^41^, ^42^, ^43^ These programs may have potential clinical applications as the movement to conduct outpatient consultations instead of inpatient consultations is growing worldwide.^44^, ^45^, ^46^ Moreover, the development of orally available drugs for cancer therapy contributes to this need. Plasma LEN concentrations have been quantified in several studies involving patients with hepatocellular carcinoma and thyroid cancer, and a therapeutic window for trough LEN concentrations in hepatocellular cancer treatment has been proposed.^7^, ^8^ However, accurately determining plasma trough LEN concentrations may be challenging due to the use of 5 days on/2 days off administration in some patients.^47^, ^48^ In the present study, plasma LEN concentration parameters, including patients with various cancer types, dosages, and usage, were used to predict plasma trough LEN concentrations, resulting in a moderate correlation between the plasma LEN concentration at the time of sampling and the predicted plasma trough LEN concentration. Thus, NONMEM may be useful in further clinical studies and future clinical applications. Interestingly, high correlation was observed in the patients with renal cell carcinoma probably because only three patients were administrated LEN everyday but not on the 5 days on/2 days off regimen. In contrast, the correlations in patients with endometrial cancer were relatively weak, possibly because no data from patients with endometrial cancer were included in the parameters used for prediction by NONMEM. The three graphs for all cancer patients, hepatocellular carcinoma, and endometrial cancer appeared to show two correlations in one graph. This may be due to factors such as differences in liver function derived from cancer type and the lag between LEN dosing times (morning or evening) and blood collection times. Taken together, these findings suggest that optimizing parameters for each cancer type including endometrial cancer, for which NONMEM parameters have not been explored, may be useful in personalized medicine.

The utility of endogenous CYP3A biomarkers was evaluated by comparing plasma LEN concentrations. In this study, the ratios of 6β‐OHC/C and 1β‐OHDCA/DCA were evaluated for their correlation with plasma LEN concentrations. The relevance between them has not been evaluated until now. Thus, this study can potentially compare them to identify the best biomarker. The concentrations of all analytes and the correlation between the substrate and metabolite indicated a similar trend compared with previous research on 18 healthy participants.^34^ However, intra‐ and inter‐ratio differences in patients largely varied, and unfortunately, no correlations were observed. In some patients, the fluctuation ranges were different between biomarkers. Specific factors impacting their levels, including the renal clearance of C, may affect these differences, but the values could not be evaluated in this study. Previously reported CYP3A activity biomarkers have been identified in strictly managed clinical studies.^25^, ^26^, ^30^, ^32^ For instance, the ratio alteration between 1β‐OHDCA and DCA was recognized under the condition of taking strong CYP3A inhibitors and urine sampling simultaneously on the same day.^26^ However, these biomarkers are difficult to manage in clinical practice; therefore, the clinical application of endogenous urinary biomarkers may be challenging.

In several biomarkers for predicting CYP activity, except for 6β‐OHC and 1β‐OHDCA, 4β‐hydroxycholesterol was reported as an endogenous plasma CYP3A biomarker.^31^, ^32^, ^33^ Compared to other biomarkers, 4β‐hydroxycholesterol has a relatively long half‐life.^31^ For patients with stable conditions without any change in medication, 4β‐hydroxycholesterol could be better for predicting CYP3A activities, but it is less important to investigate this biomarker in clinical practice compared to other biomarkers with short half‐lives because dosage adjustments should already be completed. The difficulty in predicting enzymatic activity may be specific to the CYP isoforms. Recently, a dietary CYP2D6 biomarker, 3,4‐seco‐solanidine‐3,4‐dioic acid, was identified, and its efficacy was evaluated.^49^, ^50^, ^51^ This biomarker can detect whether a patient is a CYP2D6‐poor metabolizer or not. The usefulness of CYP biomarkers may depend on CYP isoforms as the intra‐ and inter‐variety of CYP3A is larger than that of CYP2D6.^52^, ^53^ Therefore, it may be difficult to predict CYP3A activity although further investigations are required to confirm its utility.

In summary, clinically relevant concentrations of plasma LEN and endogenous urinary CYP3A activity biomarkers were investigated. The utility of NONMEM for clinical studies and future clinical applications was confirmed, whereas endogenous urinary CYP3A biomarkers are difficult to apply clinically. More accurate plasma trough LEN concentrations using NONMEM would be needed to reveal the actual timing of taking LEN and blood sampling and to optimize parameters for each cancer type. However, large amounts of patient data must be collected. Nevertheless, the utility of TDM for LEN should be investigated in more detail in future studies because the correlation between LEN concentrations and the efficacy and safety of LEN therapy could not be accurately evaluated during this study period despite monitoring LEN dosage reduction in several patients. CYP3A biomarkers may not be clinically useful; however, there is no consensus due to small sample sizes. For example, a more restricted study design is important for accurate evaluation, such as the correlation between the blood trough concentration of tacrolimus, which is primarily metabolized by CYP3A,^17^ and the concentrations of endogenous CYP3A biomarkers in urine, collected simultaneously. The LEN in urine could affect CYP3A biomarkers, but urine concentrations were not evaluated in this study. Therefore, further studies are required to determine the utility of CYP3A biomarkers accurately.

## AUTHOR CONTRIBUTIONS

Masaki Kumondai: Conceptualization, methodology, analysis, investigation, data curation, writing—original draft, visualization, and project administration. Reika Ogawa: Analysis, investigation, data curation, and writing—review & editing. Nagomi Hayashi: Analysis, data curation, and writing—review & editing. Yurika Ishida: Investigation, data curation, and writing—review & editing. Hanae Oshikiri: Investigation, data curation, and writing—review & editing. Yuji Sato: Investigation, data curation, and writing—review & editing. Masafumi Kikuchi: Methodology, analysis, and writing—review & editing. Yu Sato: Methodology and writing—review & editing. Toshihiro Sato: Methodology and writing—review & editing. Masamitsu Maekawa: Methodology, writing—review & editing, resources, and supervision. Nariyasu Mano: Methodology, writing—review & editing, resources, and supervision.

## FUNDING INFORMATION

This study was supported by the Japan Society for the Promotion of Science (grant number 22 K15333 to M. Kumondai).

## CONFLICT OF INTEREST STATEMENT

The authors declare no conflict of interest.

## ETHICS STATEMENT

The protocols were approved by the Ethical Review Board of Tohoku University (permission number 2021‐1‐928).

## PATIENT CONSENT STATEMENT

The participants provided written informed consent.

## Supporting information


Table S1.

## Data Availability

The data that support the findings of this study are available on request from the corresponding author.

## References

[1] Kudo M , Finn RS , Qin S , et al. Lenvatinib versus sorafenib in first‐line treatment of patients with unresectable hepatocellular carcinoma: a randomised phase 3 non‐inferiority trial. Lancet. 2018;391:1163‐1173. 29433850 10.1016/S0140-6736(18)30207-1

[2] Saito A , Kikuchi M , Matsumoto Y , et al. Evaluation of a capillary microsampling device for analyzing plasma lenvatinib concentration in patients with hepatocellular carcinoma. Ther Drug Monit. 2022;44:771‐776. 35863065 10.1097/FTD.0000000000001013

[3] Schlumberger M , Tahara M , Wirth LJ , et al. Lenvatinib versus placebo in radioiodine‐refractory thyroid cancer. N Engl J Med. 2015;372:621‐630. 25671254 10.1056/NEJMoa1406470

[4] Sato J , Satouchi M , Itoh S , et al. Lenvatinib in patients with advanced or metastatic thymic carcinoma (REMORA): a multicentre, phase 2 trial. Lancet Oncol. 2020;21:843‐850. 32502444 10.1016/S1470-2045(20)30162-5

[5] Taylor MH , Lee CH , Makker V , et al. Phase IB/II trial of lenvatinib plus pembrolizumab in patients with advanced renal cell carcinoma, endometrial cancer, and other selected advanced solid tumors. J Clin Oncol. 2020;38:1154‐1163. 31961766 10.1200/JCO.19.01598 PMC7145588

[6] Noda S , Morita SY , Terada T . Dose individualization of oral multikinase inhibitors for the implementation of therapeutic drug monitoring. Biol Pharm Bull. 2022;45:814‐823. 35786588 10.1248/bpb.b21-01098

[7] Nagahama M , Ozeki T , Suzuki A , et al. Association of lenvatinib trough plasma concentrations with lenvatinib‐induced toxicities in Japanese patients with thyroid cancer. Med Oncol. 2019;36:39. 30919115 10.1007/s12032-019-1263-3

[8] Noda S , Iida H , Fujimoto T , et al. Exploratory analysis of target concentration of lenvatinib in the treatment of hepatocellular carcinoma. Cancer Chemother Pharmacol. 2021;88:281‐288. 33928425 10.1007/s00280-021-04286-2

[9] Iwasaki H , Toda S , Murayama D , Kato S , Matsui A . Relationship between adverse events associated with lenvatinib treatment for thyroid cancer and patient prognosis. Mol Clin Oncol. 2021;14:28. 33414909 10.3892/mco.2020.2190 PMC7783723

[10] Nakamichi S , Nokihara H , Yamamoto N , et al. A phase 1 study of lenvatinib, multiple receptor tyrosine kinase inhibitor, in Japanese patients with advanced solid tumors. Cancer Chemother Pharmacol. 2015;76:1153‐1161. 26530955 10.1007/s00280-015-2899-0 PMC4648947

[11] Daly AK . Significance of the minor cytochrome P450 3A isoforms. Clin Pharmacokinet. 2006;45:13‐31. 16430309 10.2165/00003088-200645010-00002

[12] Klyushova LS , Perepechaeva ML , Grishanova AY . The role of CYP3A in health and disease. Biomedicine. 2022;10:2686. 10.3390/biomedicines10112686 PMC9687714 36359206

[13] Zanger UM , Schwab M . Cytochrome P450 enzymes in drug metabolism: regulation of gene expression, enzyme activities, and impact of genetic variation. Pharmacol Ther. 2013;138:103‐141. 23333322 10.1016/j.pharmthera.2012.12.007

[14] Mikus G , Foerster KI . Role of CYP3A4 in kinase inhibitor metabolism and assessment of CYP3A4 activity. Transl Cancer Res. 2017;6:S1592‐S1599.

[15] Hendriks DFG , Vorrink SU , Smutny T , et al. Clinically relevant cytochrome P450 3A4 induction mechanisms and drug screening in three‐dimensional spheroid cultures of primary human hepatocytes. Clin Pharmacol Ther. 2020;108:844‐855. 32320483 10.1002/cpt.1860

[16] Kumondai M , Gutierrez Rico EM , Hishinuma E , et al. Functional characterization of 40 CYP3A4 variants by assessing midazolam 1′‐hydroxylation and testosterone 6beta‐hydroxylation. Drug Metab Dispos. 2021;49:212‐220. 33384383 10.1124/dmd.120.000261

[17] Kumondai M , Kikuchi M , Mizuguchi A , et al. Therapeutic drug monitoring of blood sirolimus and tacrolimus concentrations for polypharmacy management in a lymphangioleiomyomatosis patient taking two cytochrome P450 3A inhibitors. Tohoku J Exp Med. 2023;260:29‐34. 36858510 10.1620/tjem.2023.J016

[18] Huang SM , Strong JM , Zhang L , et al. New era in drug interaction evaluation: US Food and Drug Administration update on CYP enzymes, transporters, and the guidance process. J Clin Pharmacol. 2008;48:662‐670. 18378963 10.1177/0091270007312153

[19] Elens L , van Gelder T , Hesselink DA , Haufroid V , van Schaik RH . CYP3A4*22: promising newly identified CYP3A4 variant allele for personalizing pharmacotherapy. Pharmacogenomics. 2013;14:47‐62. 23252948 10.2217/pgs.12.187

[20] Otsuki A , Kumondai M , Kobayashi D , et al. Plasma venetoclax concentrations in patients with acute myeloid leukemia treated with CYP3A4 inhibitors. Yakugaku Zasshi. 2024;144:775‐779. 38945852 10.1248/yakushi.24-00018

[21] Inoue K , Mizuo H , Kawaguchi S , Fukuda K , Kusano K , Yoshimura T . Oxidative metabolic pathway of lenvatinib mediated by aldehyde oxidase. Drug Metab Dispos. 2014;42:1326‐1333. 24914245 10.1124/dmd.114.058073

[22] Vavrova K , Indra R , Pompach P , Heger Z , Hodek P . The impact of individual human cytochrome P450 enzymes on oxidative metabolism of anticancer drug lenvatinib. Biomed Pharmacother. 2022;145:112391. 34847475 10.1016/j.biopha.2021.112391

[23] Shumaker RC , Aluri J , Fan J , Martinez G , Thompson GA , Ren M . Effect of rifampicin on the pharmacokinetics of lenvatinib in healthy adults. Clin Drug Investig. 2014;34:651‐659. 10.1007/s40261-014-0217-y PMC4143598 25022720

[24] Ozeki T , Nagahama M , Fujita K , et al. Influence of CYP3A4/5 and ABC transporter polymorphisms on lenvatinib plasma trough concentrations in Japanese patients with thyroid cancer. Sci Rep. 2019;9:5404. 30931962 10.1038/s41598-019-41820-y PMC6443943

[25] Hayes MA , Li XQ , Gronberg G , Diczfalusy U , Andersson TB . CYP3A specifically catalyzes 1beta‐hydroxylation of deoxycholic acid: characterization and enzymatic synthesis of a potential novel urinary biomarker for CYP3A activity. Drug Metab Dispos. 2016;44:1480‐1489. 27402728 10.1124/dmd.116.070805

[26] Li XQ , Thelingwani RS , Bertilsson L , Diczfalusy U , Andersson TB , Masimirembwa C . Evaluation of 1β‐hydroxylation of deoxycholic acid as a non‐invasive urinary biomarker of CYP3A activity in the assessment of inhibition‐based drug‐drug interaction in healthy volunteers. J Pers Med. 2021;11:457. 34073662 10.3390/jpm11060457 PMC8224742

[27] Magliocco G , Desmeules J , Bosilkovska M , Thomas A , Daali Y . The 1β‐hydroxy‐deoxycholic acid to deoxycholic acid urinary metabolic ratio: an endogenous CYP3A metric? Eur J Clin Pharmacol. 2021;11:150. 10.3390/jpm11020150 PMC7923269 33672438

[28] Joellenbeck L , Qian Z , Zarba A , Groopman JD . Urinary 6 beta‐hydroxycortisol/cortisol ratios measured by high‐performance liquid chromatography for use as a biomarker for the human cytochrome P‐450 3A4. Cancer Epidemiol Biomarkers Prev. 1992;1:567‐572. 1302569

[29] Rais N , Hussain A , Chawla YK , Kohli KK . Association between urinary 6beta‐hydroxycortisol/cortisol ratio and CYP3A5 genotypes in a normotensive population. Exp Ther Med. 2013;5:527‐532. 23404385 10.3892/etm.2012.842 PMC3570147

[30] Bergström H , Lindahl A , Warnqvist A , Diczfalusy U , Ekström L , Björkhem‐Bergman L . Studies on CYP3A activity during the menstrual cycle as measured by urinary 6β‐hydroxycortisol/cortisol. Pharmacol Res Perspect. 2021;9:e00884. 34664787 10.1002/prp2.884 PMC8525181

[31] Diczfalusy U , Kanebratt KP , Bredberg E , Andersson TB , Böttiger Y , Bertilsson L . 4β‐Hydroxycholesterol as an endogenous marker for CYP3A4/5 activity. Stability and half‐life of elimination after induction with rifampicin. Brit J Clin Pharmaco. 2009;67:38‐43. 10.1111/j.1365-2125.2008.03309.x PMC2668082 19006545

[32] Gravel S , Chiasson JL , Gaudette F , Turgeon J , Michaud V . Use of 4β‐hydroxycholesterol plasma concentrations as an endogenous biomarker of CYP3A activity: clinical validation in individuals with type 2 diabetes. Clin Pharmacol Ther. 2019;106:831‐840. 31002385 10.1002/cpt.1472

[33] Penzak SR , Rojas‐Fernandez C . 4β‐Hydroxycholesterol as an endogenous biomarker for CYP3A activity: literature review and critical evaluation. J Clin Pharmacol. 2019;59:611‐624. 30748026 10.1002/jcph.1391

[34] Kumondai M , Maekawa M , Hishinuma E , et al. Development of a simultaneous liquid chromatography‐tandem mass spectrometry analytical method for urinary endogenous substrates and metabolites for predicting cytochrome P450 3A4 activity. Biol Pharm Bull. 2023;46:455‐463. 36858575 10.1248/bpb.b22-00840

[35] Gupta A , Jarzab B , Capdevila J , Shumaker R , Hussein Z . Population pharmacokinetic analysis of lenvatinib in healthy subjects and patients with cancer. Br J Clin Pharmacol. 2016;81:1124‐1133. 26879594 10.1111/bcp.12907 PMC4876185

[36] Harding SD , Sharman JL , Faccenda E , et al. The IUPHAR/BPS guide to PHARMACOLOGY in 2018: updates and expansion to encompass the new guide to IMMUNOPHARMACOLOGY. Nucleic Acids Res. 2018;46:D1091‐D1106. 29149325 10.1093/nar/gkx1121 PMC5753190

[37] Alexander SPH , Fabbro D , Kelly E , et al. The concise guide to PHARMACOLOGY 2023/24: catalytic receptors. Br J Pharmacol. 2023;180:S241‐S288. 38123155 10.1111/bph.16180

[38] Alexander SPH , Fabbro D , Kelly E , et al. The concise guide to PHARMACOLOGY 2023/24: enzymes. Br J Pharmacol. 2023;180:S289‐S373. 38123154 10.1111/bph.16181

[39] Keizer RJ , Karlsson MO , Hooker A . Modeling and simulation workbench for NONMEM: tutorial on Pirana, PsN, and Xpose. CPT Pharmacometrics Syst Pharmacol. 2013;2:e50. 23836189 10.1038/psp.2013.24 PMC3697037

[40] Clemente‐Bautista S , Troconiz IF , Segarra‐Canton O , et al. The effect of polymorphisms and other biomarkers on infliximab exposure in paediatric inflammatory bowel disease: development of a population pharmacokinetic model. Paediatr Drugs. 2024;26:331‐346. doi:10.1007/s40272-024-00621-1 38507036

[41] Duong A , Simard C , Williamson D , Marsot A . Tobramycin a priori dosing regimens based on PopPK model simulations in critically ill patients: are they transferable? Ther Drug Monit. 2023;45:616‐622. 36917735 10.1097/FTD.0000000000001091

[42] Yoon S , Jin BH , Kim CO , Park K , Park MS , Chae D . Pharmacokinetic modeling of bepotastine for determination of optimal dosage regimen in pediatric patients with allergic rhinitis or urticaria. Pharmaceutics. 2024;16:334. 38543228 10.3390/pharmaceutics16030334 PMC10975960

[43] Zhang C , Jiang L , Hu K , et al. Effects of aripiprazole on olanzapine population pharmacokinetics and initial dosage optimization in schizophrenia patients. Neuropsychiatr Dis Treat. 2024;20:479‐490. 38469209 10.2147/NDT.S455183 PMC10925492

[44] Bauer RJ . NONMEM tutorial part I: description of commands and options, with simple examples of population analysis. CPT Pharmacometrics Syst Pharmacol. 2019;8:525‐537. 31056834 10.1002/psp4.12404 PMC6709426

[45] Bauer RJ . NONMEM tutorial part II: estimation methods and advanced examples. CPT Pharmacometrics Syst Pharmacol. 2019;8:538‐556. 31044558 10.1002/psp4.12422 PMC6709422

[46] Wang C , Allegaert K , Peeters MY , Tibboel D , Danhof M , Knibbe CA . The allometric exponent for scaling clearance varies with age: a study on seven propofol datasets ranging from preterm neonates to adults. Br J Clin Pharmacol. 2014;77:149‐159. 23772816 10.1111/bcp.12180 PMC3895356

[47] Iwamoto H , Suzuki H , Shimose S , et al. Weekends‐off lenvatinib for unresectable hepatocellular carcinoma improves therapeutic response and tolerability toward adverse events. Cancer. 2020;12:1010. 10.3390/cancers12041010 PMC7226076 32325921

[48] Sunaga N , Miura Y , Sakurai R , et al. Sustained antitumor response to lenvatinib with weekend‐off and alternate‐day administration in chemotherapy‐refractory thymic carcinoma: a case report. Anti‐Cancer Drugs. 2023;34:605‐608. 36729850 10.1097/CAD.0000000000001474

[49] Behrle AC , Douglas J , Leeder JS , van Haandel L . Isolation and identification of 3,4‐seco‐solanidine‐3,4‐dioic acid (SSDA) as a urinary biomarker of cytochrome P450 2D6 (CYP2D6) activity. Drug Metab Dispos. 2022;10:1342‐1351. 10.1124/dmd.122.000957 PMC9513856 35878926

[50] Kiiski JI , Neuvonen M , Kurkela M , et al. Solanidine is a sensitive and specific dietary biomarker for CYP2D6 activity. Hum Genomics. 2024;18:11. 38303026 10.1186/s40246-024-00579-8 PMC10835938

[51] Wollmann BM , Smith RL , Kringen MK , Ingelman‐Sundberg M , Molden E , Storset E . Evidence for solanidine as a dietary CYP2D6 biomarker: significant correlation with risperidone metabolism. Br J Clin Pharmacol. 2024;90:740‐747. 36960588 10.1111/bcp.15721

[52] Chiba K , Kato M , Ito T , Suwa T , Sugiyama Y . Inter‐individual variability of in vivo CYP2D6 activity in different genotypes. Drug Metab Pharmacokinet. 2012;27:405‐413. 22277677 10.2133/dmpk.dmpk-11-rg-078

[53] Fujino C , Sanoh S , Katsura T . Variation in expression of cytochrome P450 3A isoforms and toxicological effects: endo‐ and exogenous substances as regulatory factors and substrates. Biol Pharm Bull. 2021;44:1617‐1634. 34719640 10.1248/bpb.b21-00332

